# Supplementary material for: Improved Reverse Transcription Loop-Mediated Isothermal Amplification (RT-LAMP) for the Rapid and Sensitive Detection of Yam mosaic virus
Source: Viruses. 2023 Jul 21;15(7):1592. doi: 10.3390/v15071592 (PMC10383231; doi:10.3390/v15071592)
Supplement: Supplementary file 1 [file viruses-15-01592-s001.zip › Supplementary figure 1.pdf]

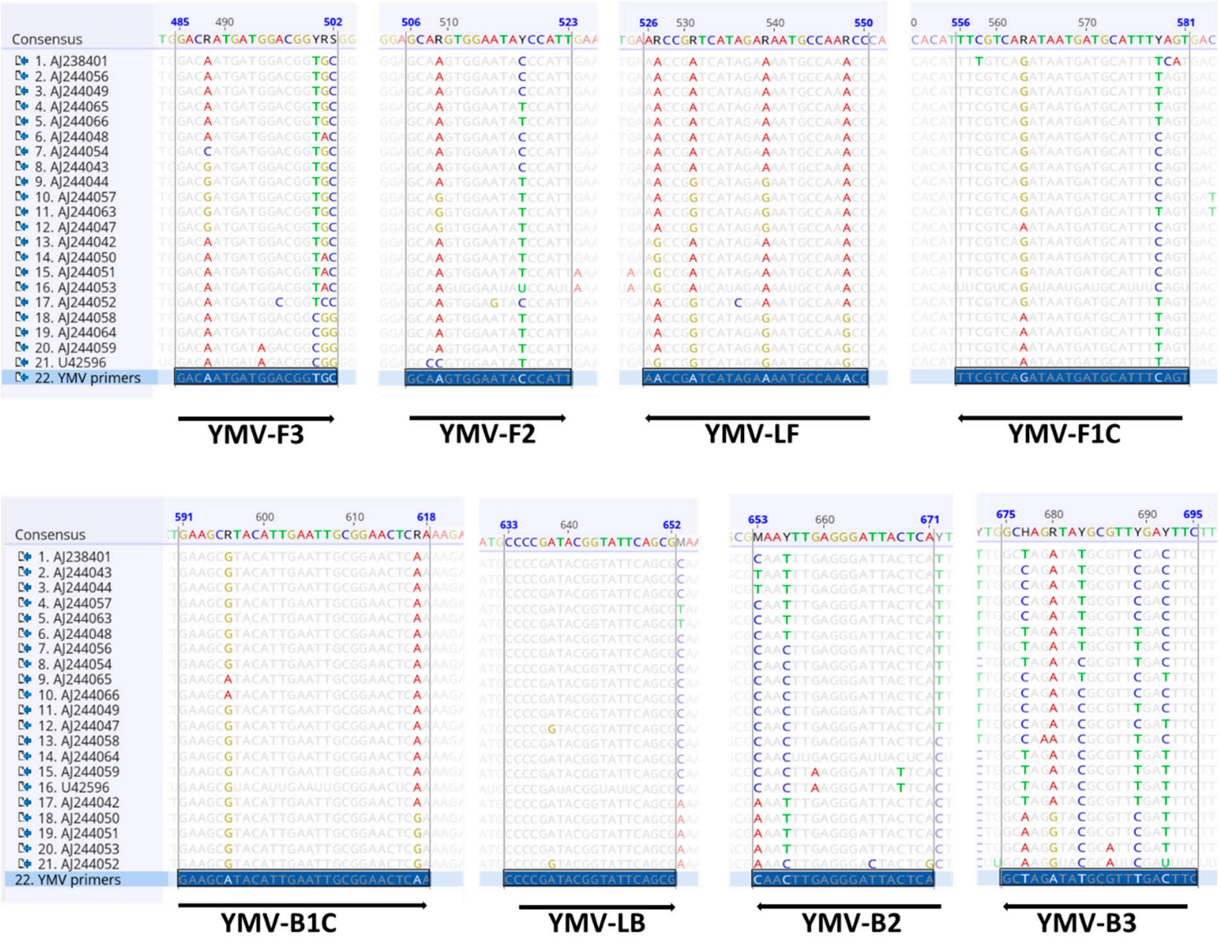

Figure S1: Alignment of YMV coat protein sequences, highlighting the YMV LAMP primers by Nkere et al., [23]
